# Effects of the preparation method on the structure and the visible-light photocatalytic activity of Ag_2_CrO_4_

**DOI:** 10.3762/bjnano.5.77

**Published:** 2014-05-19

**Authors:** Difa Xu, Shaowen Cao, Jinfeng Zhang, Bei Cheng, Jiaguo Yu

**Affiliations:** 1 State Key Laboratory of Advanced Technology for Materials Synthesis and Processing, Wuhan University of Technology, Wuhan 430070, P. R. China; 2 Department of Biological and Environmental Science, Changsha University, Changsha 410022, P.R. China

**Keywords:** microemulsion method, nanoparticles, photocatalysis, photocatalytic activity, silver chromate, visible-light-driven

## Abstract

Silver chromate (Ag_2_CrO_4_) photocatalysts are prepared by microemulsion, precipitation, and hydrothermal methods, in order to investigate the effect of preparation methods on the structure and the visible-light photocatalytic activity. It is found that the photocatalytic activity of the prepared Ag_2_CrO_4_was highly dependent on the preparation methods. The sample prepared by microemulsion method exhibits the highest photocatalytic efficiency on the degradation of methylene blue (MB) under visible-light irradiation. The enhanced photocatalytic activity could be ascribed to the smaller particle size, higher surface area, relatively stronger light absorption, and blue-shift absorption edge, which result in the adsorption of more MB molecules, a shorter diffusion process of more photogenerated excitons, and a stronger oxidation ability of the photogenerated holes. Considering the universalities of microemulsion, precipitation, and hydrothermal methods, this work may also provide a prototype for the comparative study of semiconductor based photocatalysis for water purification and environmental remediation.

## Introduction

Semiconductor photocatalysis has been considered as a potential solution to the worldwide energy shortage and for counteracting environmental degradation [1–5]. Numerous efforts have been made to develop efficient and stable photocatalysts during the past decades. TiO_2_ is most widely studied because of its low cost, non-toxicity, high efficiency and long-time photostability [6–11]. However, due to its large band gap of about 3.2 eV, TiO_2_ is only active in the ultraviolet (UV) region that corresponds to 3–4% of the solar light. Therefore, the development of visible-light-driven photocatalysts has received considerable attention as visible light (400–800 nm) is abundant in the solar spectrum [12–16]. Some semiconductors such as BiVO_4_ [17–19], Bi_2_O_3_ [20–21], Fe_2_O_3_ [22–25], and Cu_2_O [26–28] have been developed as photocatalysts with visible-light activities. Recently, a family of Ag-based salts, including Ag_3_PO_4_ [29–30], Ag_2_CO_3_ [31–32], AgVO_3_ [33–34], AgGaO_2_ [35–36], has attracted particular interests due to their ability to split water, as well as decompose organic contaminants both in air and aqueous solution. However, Ag_2_CrO_4_ is neglected although it has been explored as cathode for lithium cells in early years [37–39]. Actually, the band gap of Ag_2_CrO_4_ is narrow enough (about 1.75 eV) to obtain strong absorption in visible-light region [40], and thus may enable it to perform excellent visible-light photocatalytic activity. However, up to now, the photocatalytic studies on Ag_2_CrO_4_ are still limited with only few reports [40–42].

So far, several methods have been employed for the preparation of Ag_2_CrO_4_ crystals, such as precipitation [43], reversed-micellar [44], hydrothermal [45], sonochemical [41], and template methods [46]. It is known that the photocatalytic activity of semiconductor photocatalysts relies heavily on their structures, which are commonly determined by the preparation methods [47–49]. Nevertheless, to our knowledge, there is no comparative study about the effect of the preparation methods on the structure and photocatalytic performance of Ag_2_CrO_4_. Herein, for the first time, special attention is paid to evaluate the effect of preparation methods on the structure and visible-light photocatalytic activity of Ag_2_CrO_4_. Microemulsion, precipitation, and hydrothermal methods are selected for preparing Ag_2_CrO_4_ photocatalysts, as they are simple, efficient, and extensively used preparation methods for semiconductor crystals. In this case, the present work can also provide a prototype for comparative study of other semiconductor photocatalysts. The performance of the developed Ag_2_CrO_4_ is evaluated by the photocatalytic degradation of methylene blue under visible-light irradiation. The effect of the three preparation methods on the structure, optical properties and photocatalytic activity are investigated and discussed.

## Results and Discussion

### Phase structure and morphology

**Figure 1:**
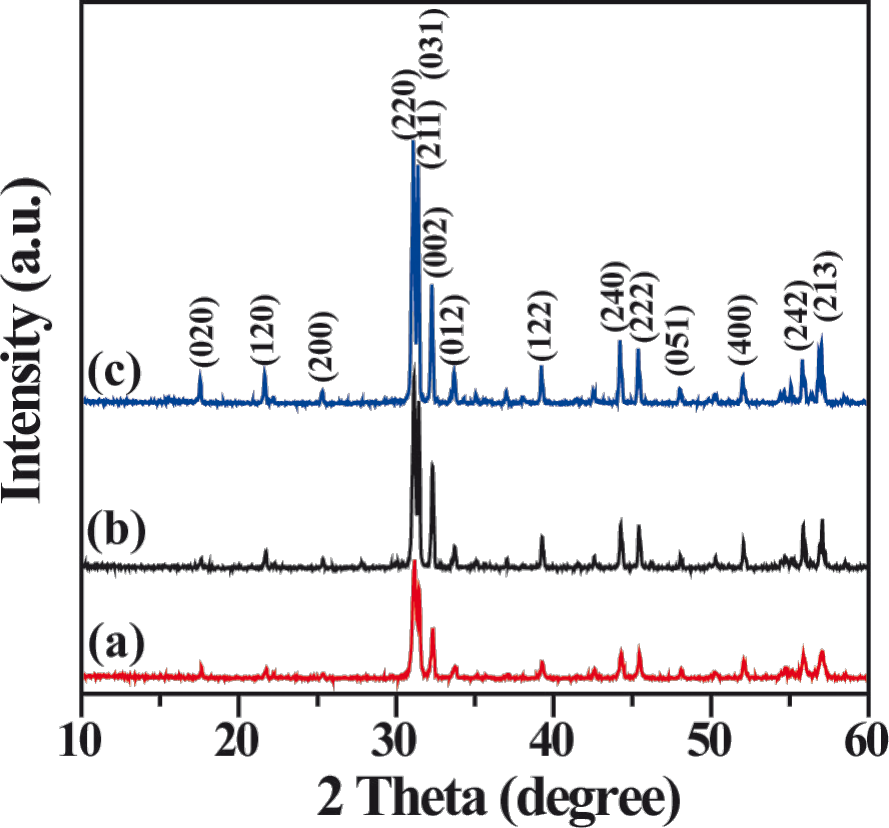
XRD patterns of Ag_2_CrO_4_ samples prepared by different methods: (a) microemulsion, (b) precipitation, and (c) hydrothermal.

The X-ray diffraction (XRD) patterns are shown in Figure 1. All the diffraction peaks for the S-M, S-P, and S-H samples respectively prepared by microemulsion, precipitation, and hydrothermal methods can be indexed to the orthorhombic phase of Ag_2_CrO_4_ (JCPDS No. 26-0952). It is noted that the diffraction peaks of S-H sample exhibit the highest intensity (Figure 1c), resulting from the improved crystallinity of Ag_2_CrO_4_ promoted by hydrothermal reaction at the high temperature and pressure conditions [45]. While the crystal growth in microemulsion is restricted due to the effect of steric barrier [50–51], it is not surprising that the lowest intensity is observed for the diffraction peaks of S-M sample (Figure 1a).

**Figure 2:**
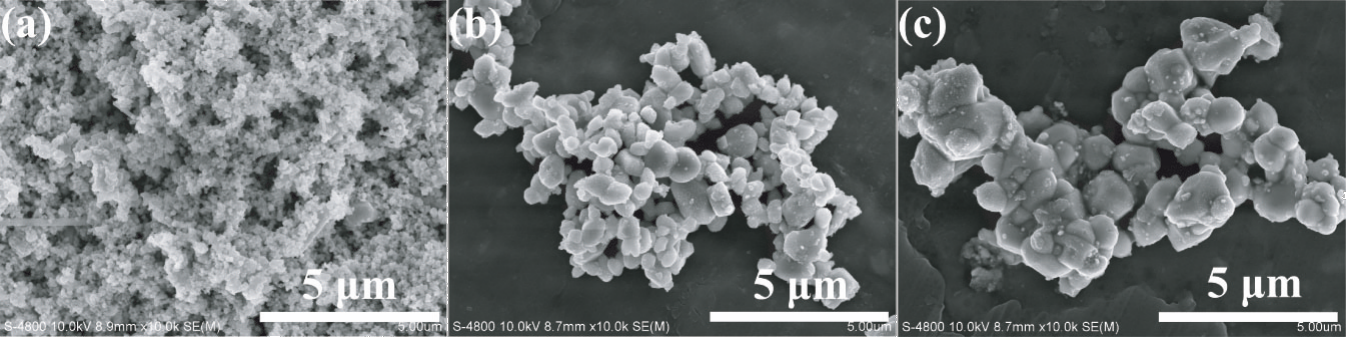
SEM images of Ag_2_CrO_4_ samples obtained from different methods: (a) microemulsion, (b) precipitation, and (c) hydrothermal.

**Figure 3:**
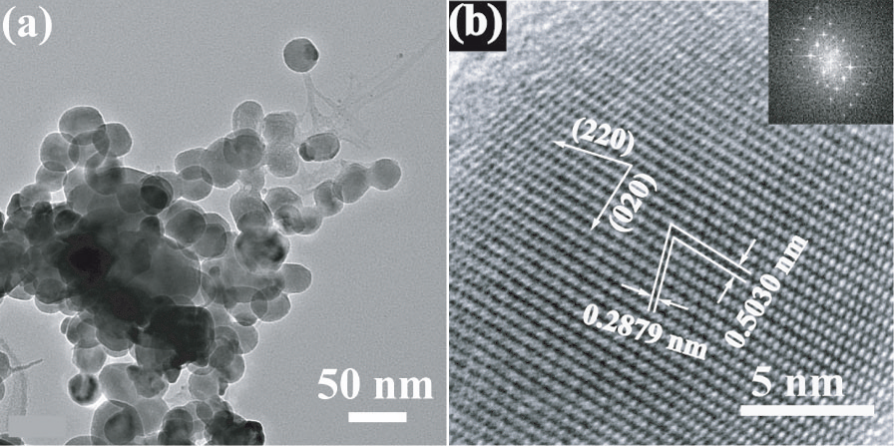
TEM (a) and HRTEM (b) images of Ag_2_CrO_4_ sample prepared by microemulsion method. The inset of (b) is the corresponding FFT image.

Scanning electron microscope (SEM) images are taken to directly analyze the structure of the prepared Ag_2_CrO_4_ samples and particularly the effect of the preparation methods on the morphology changes. Figure 2 indicates that the S-M sample has a more homogenous morphology, and the average particle size is much smaller than those of S-P (ca. 800 nm) and S-H (ca. 1.2 μm) samples. For an in-depth investigation of the morphology and particle size of the S-M sample, transmission electron microscopy (TEM) observation is carried out. As shown in Figure 3a, the S-M sample is composed of nanoparticles with an average particle size of about 30 nm. The high-resolution transmission electron microscopy (HRTEM) image in Figure 3b clearly shows the lattice fringes with *d* spacings of 0.503 and 0.288 nm, which can be assigned to the (020) and (220), respectively, crystal planes of orthorhombic Ag_2_CrO_4_. The corresponding fast Fourier transform (FFT) image suggests a single-crystalline nature. This also indicates that the S-M sample is well-crystallized, although its XRD pattern exhibits a relatively lower intensity (Figure 1a). In our experiment, a dynamically stable and isotropic W/O reverse microemulsion system is established by using cyclohexane as oil phase, Triton X-100 as surfactant, and *n*-hexanol as co-surfactant, respectively. Hence a more homogeneous morphology and smaller particle size of Ag_2_CrO_4_ can be achieved since the precipitation reaction is restricted in nanosized water droplets, which are dispersed as liquid entities in a continuous oil media and act as nanoreactors for the synthesis of nanoparticles [52–55]. Furthermore, Triton X-100 serves as a nonionic surfactant in the W/O reverse microemulsion system to avoid the introduction of ionic impurities. These results suggest that the microemulsion method is superior for preparing Ag_2_CrO_4_ nanoparticles with homogenous distribution, as compared to the precipitation and hydrothermal methods.

### Brunauer–Emmett–Teller (BET) surface area and pore size distributions

**Figure 4:**
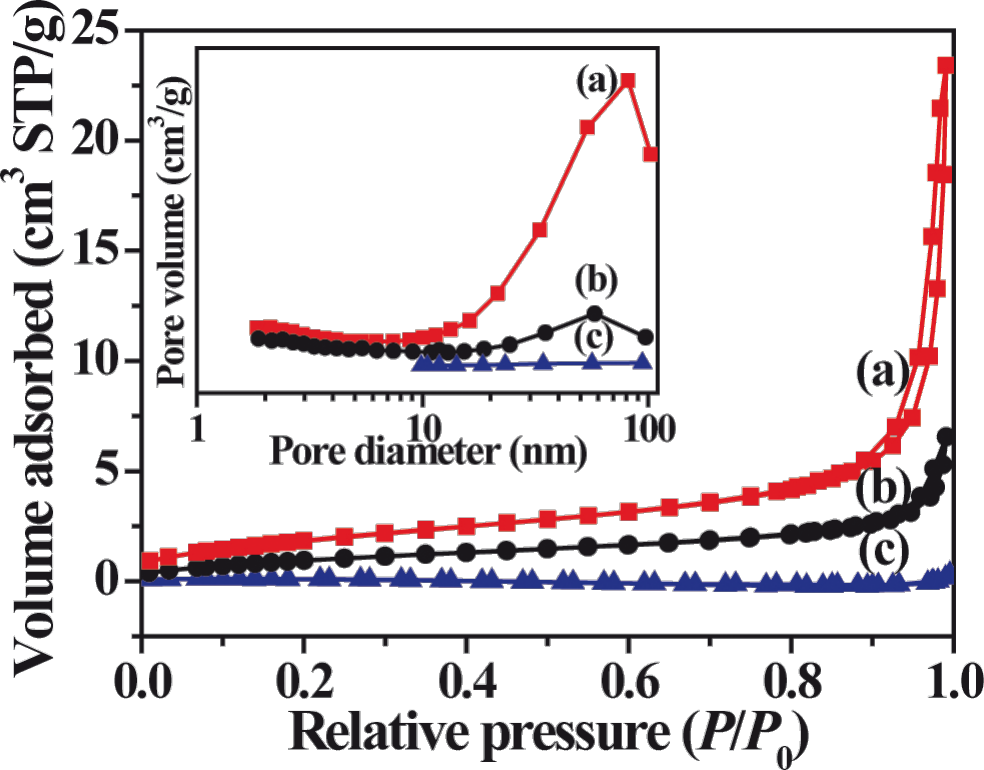
Nitrogen adsorption-desorption isotherms and corresponding pore size distribution curves (inset) of Ag_2_CrO_4_ samples prepared by different methods: (a) microemulsion, (b) precipitation, and (c) hydrothermal.

Figure 4 shows the nitrogen adsorption–desorption isotherms and the corresponding pore size distributions of the as-prepared Ag_2_CrO_4_ photocatalysts. According to the Brunauer–Deming–Deming–Teller classification, the isotherms of all Ag_2_CrO_4_ samples are of type IV, indicating the presence of mesopores (2–50 nm) [56–58]. Moreover, the shapes of the hysteresis loops are of type H3 at the high relative pressure range from 0.8 to 1.0, which suggests the formation of large mesopores and macropores [56]. The pore size distributions (inset of Figure 4) are very broad, further confirming the presence of large mesopores and macropores. Considering the absence of a pore structure inside the individual nanoparticles on the basis of SEM and TEM results, these pores can be related to the pores between the aggregated Ag_2_CrO_4_ particles. The Ag_2_CrO_4_ samples show decreasing specific surface areas in the sequence S-M, S-P, and S-H, which are listed in Table 1. This is because the S-M sample has the smallest particle size, whereas the S-H sample has the largest particle size. Usually, photocatalysts with higher specific surface areas are beneficial for the enhancement of photocatalytic performance by facilitating the absorption of pollutants for degradation.

**Table 1:** Physical properties and photocatalytic performance of as-prepared samples.

sample	preparation method	particle size (nm)	*S*_BET_^a^ (m^2^/g)	band gap (eV)	rate constant *k* (min^−1^)

S-M	microemulsion	30	7.0	1.85	0.033
S-P	precipitation	800	4.0	1.82	0.020
S-H	hydrothermal	1200	0.3	1.76	0.015

^a^BET specific surface area.

### UV–vis spectroscopy measurements

**Figure 5:**
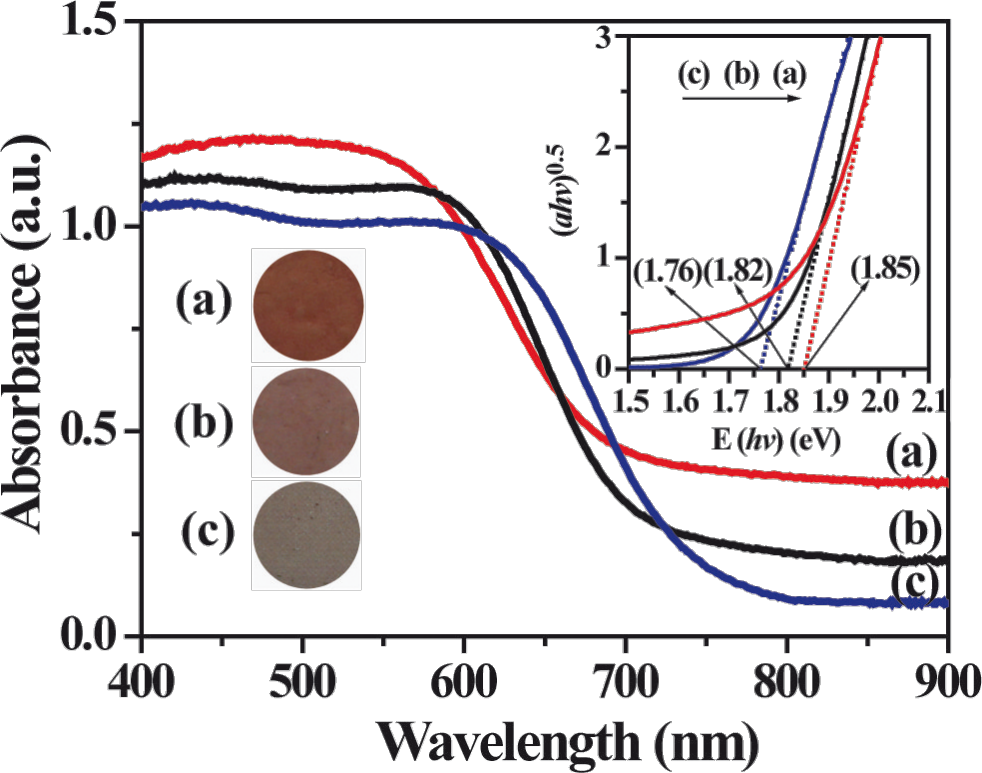
UV–visible diffuse reflectance spectra, the calculated band gaps (upper right inset) and the corresponding colours (lower left inset) of Ag_2_CrO_4_ samples prepared by different methods: (a) microemulsion, (b) precipitation, and (c) hydrothermal.

A comparison of UV–vis diffuse reflectance spectra (DRS) and the corresponding colours of the Ag_2_CrO_4_ samples are displayed in Figure 5. An enhanced absorption of visible light in the range of 400–600 nm and 700–900 nm can be observed for the S-M sample (Figure 5a), as compared to that for the S-P sample (Figure 5c), which may be attributed to the increased intensity of the scattered light in the sample with smaller particle size [59]. Moreover, there is an obvious blue shift of the absorption edge for the S-M sample, which should be explained in terms of the small size effect [60–61]. In contrast, a weaker light absorption and red shift of the absorption edge are observed for the S-H sample (Figure 5b), because it has the largest particle size among the three samples. The indirect band gaps of the Ag_2_CrO_4_ samples are calculated according to the Kubelka–Munk (KM) method by the following equation [62]:

[1]
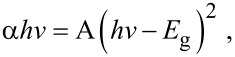


where α is the absorption coefficient, *h*ν is the photon energy, *E*_g_ is the indirect band gap, and A is a constant. As shown in the inset of Figure 5, the calculated band gap energies of the S-M, S-P and S-H samples are 1.85, 1.82 and 1.76 eV, respectively (Table 1). In spite of the little difference of the band gaps, it is clear that all the three Ag_2_CrO_4_ samples exhibit an excellent visible-light response for photocatalytic applications.

### Calculation

**Figure 6:**
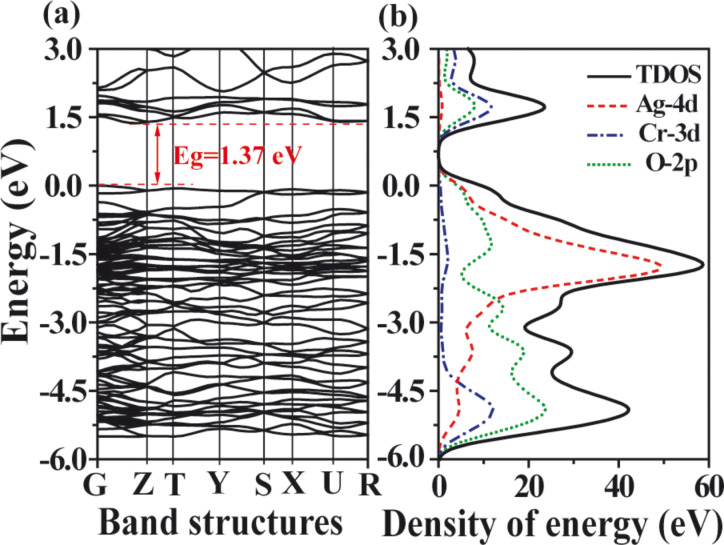
Band structure plots (a) and density of states (b) for Ag_2_CrO_4_.

Theoretically, the band structure of the Ag_2_CrO_4_ is also calculated by density function theory (DFT) (Figure 6). As shown in the band structure plots, the calculated band gap energy of Ag_2_CrO_4_ is 1.37 eV, which is lower than the experimental values, which is due to the well-known limitation of DFT calculation [63–64]. The electronic structure of Ag_2_CrO_4_ indicates that the valence band mainly consists of occupied Ag 4d and O 2p orbitals, and the conduction band mainly comes from the empty Cr 3d orbital, which means that Cr makes an important contribution to the bottom of the conduction band. It has been found that Ag is one of the elements that are able to form a valence band position higher than the O 2p orbital [65]. It has been demonstrated that Cr has the potential ability to lower down the bottom of the conduction band [40,66]. Thereby the synergistic effect of Ag and Cr elements results in the narrow band gap of Ag_2_CrO_4_. The calculated results also show that the top of the valence band is at the G point but the bottom of the conduction band is near the Z point, which confirms that Ag_2_CrO_4_ has an indirect band gap structure. These results indicate that Ag_2_CrO_4_ can potentially serve as a visible-light-driven photocatalyst.

### Photocatalytic activity

**Figure 7:**
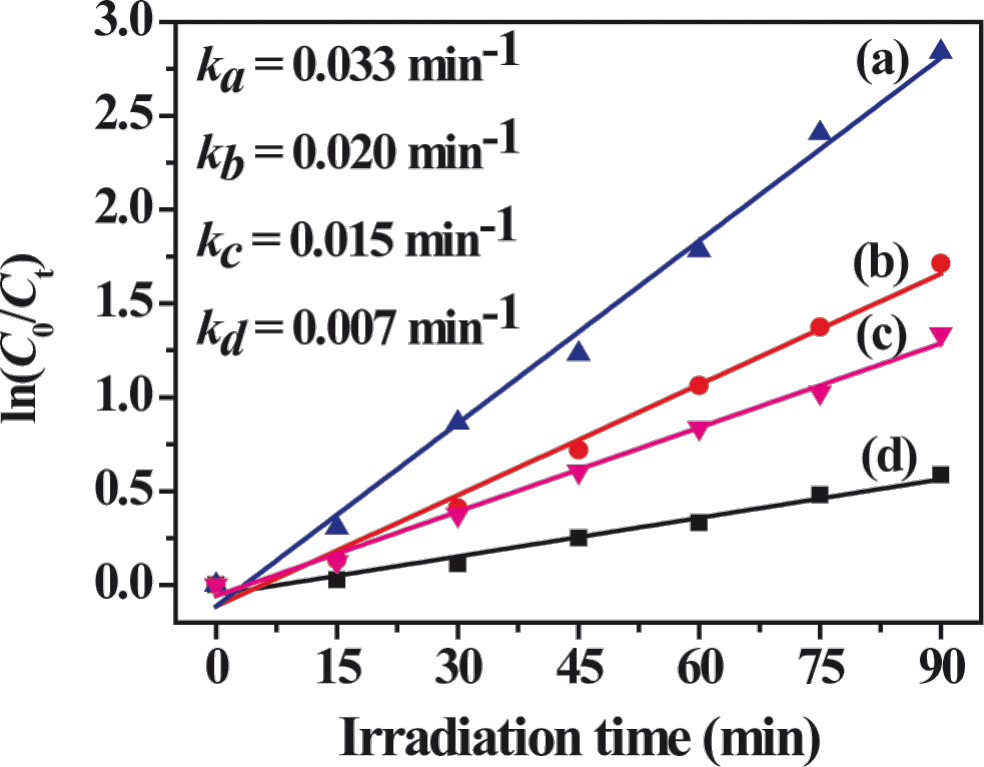
Photocatalytic degradation of MB aqueous solution over Ag_2_CrO_4_ samples prepared by (a) microemulsion, (b) precipitation, and (c) hydrothermal methods, and (d) P25 sample under visible-light irradiation.

We have measured the zeta potential of Ag_2_CrO_4_ as −15.8 mV at pH 6.8, suggesting that it is electronegative in neutral solutions. Since MB is a cationic dye, it can be easily adsorbed on the surface of Ag_2_CrO_4_ through electrostatic interaction. Therefore, the photocatalytic activity of the as-prepared Ag_2_CrO_4_ samples is evaluated through MB degradation under visible-light irradiation. Without any photocatalyst, no obvious MB degradation is observed under visible-light irradiation. For comparison, P25 (commercial TiO_2_, Degussa, Germany) is also used as a reference. Figure 7 shows that all Ag_2_CrO_4_ samples exhibit a much better photocatalytic performance than P25 in the MB degradation. In particular, the S-M Ag_2_CrO_4_ sample shows the highest activity with a rate constant of 0.033 min^−1^, and MB is almost completely degraded within 90 min. The S-P and S-H samples exhibit a lower activity with rate constants of 0.020 and 0.012 min^−1^, respectively. P25 is a mixed-phase TiO_2_ containing 25% rutile, whit a band gap of 3.0 eV, which results in a weak visible-light absorption up to about 413 nm. Therefore, P25 still shows some photocatalytic activity under visible-light irradiation. But it is not surprising that the rate constant for P25 is only 0.007 min^−1^ because the other phase anatase (75%) is not active in the visible region.

Generally, the photocatalytic degradation of dyes in water is mainly attributed to the photogenerated holes, and the active oxygen species, including superoxide radicals (O_2_^•−^) and hydroxyl radicals (OH^•^). In order to understand the possible mechanism of the photocatalytic degradation of MB over Ag_2_CrO_4_, we have determined the CB and VB position at the point of zero charge by a widely accepted approach based on the following equation [67–68]:

[2]
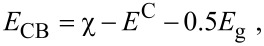


where *E*_CB_ is the CB edge potential, χ is the absolute electronegativity of the semiconductor. *E*^C^ is the energy of free electrons on the hydrogen scale (ca. 4.5 eV), and *E*_g_is the band gap of the semiconductor. Accordingly, the CB energy level of Ag_2_CrO_4_ is calculated to be ca. 0.46 eV (vs NHE), which is less negative than the O_2_/O_2_^•−^ potential; and the VB energy level of Ag_2_CrO_4_ is calculated to be ca. 2.26 eV (vs NHE), which is less positive than OH^•^/OH^−^ potential [69]. As a result, the photogenerated electrons on the CB of Ag_2_CrO_4_ are not able to reduce the adsorbed O_2_ to yield O_2_^•−^, meanwhile the photogenerated holes on the VB of Ag_2_CrO_4_ also can not oxidize H_2_O to form OH^•^ due to their insufficient reduction (for electron) and oxidation ability (for hole). In addition, the energy level of the lowest unoccupied molecular orbital (LUMO) and the highest occupied molecular orbital (HOMO) of MB are reported as ca. −0.25 and 1.6 eV [70–71], respectively. As such, the direct decomposition of MB molecules by the photogenerated holes on the VB of Ag_2_CrO_4_ is expected, since the VB of Ag_2_CrO_4_ is more positive than the HOMO of MB. Therefore, we assume that the main active species for the photocatalytic degradation of MB over Ag_2_CrO_4_ should be photogenerated holes.

The highest photocatalytic efficiency for the S-M sample is attributed to several major factors. First, the S-M sample has the highest surface area. Its photocatalytic efficiency is improved by adsorbing more MB molecules for a more efficient interaction between MB and Ag_2_CrO_4_. Second, the smaller particle size of the S-M sample can shorten the diffusion process of photogenerated electrons and holes to the surface of Ag_2_CrO_4_, thus reducing the rate of recombination [72]. Third, the enhanced visible-light absorption in the range of 400–600 nm for the S-M sample (Figure 5) can allow for a more efficient utilization of the solar energy to generate more electrons and holes and to further promote the catalytic process. Finally, the relatively wider band gap, compared to those of the S-P and S-H samples, calculated from the obvious blue-shift absorption edge of the S-M sample (Figure 5) can lead to a higher redox potential, thereby resulting in a stronger oxidation ability of the photogenerated holes [73–74]. Overall the results suggest that the photocatalytic efficiency of the Ag_2_CrO_4_ samples is influenced by the surface area, particle size and optical property, which originate from the different structure caused by different preparation methods.

**Figure 8:**
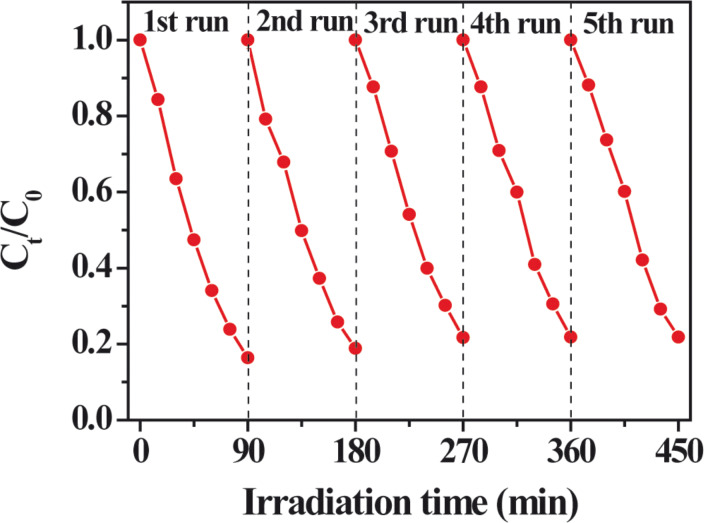
Cycling test of the photocatalytic degradation under visible-light irradiation of a MB aqueous solution in the presence of the Ag_2_CrO_4_ sample prepared by microemulsion method.

We further explore the photocatalytic stability of the S-M Ag_2_CrO_4_ sample by a cycling test of photocatalytic degradation of a MB aqueous solution under visible-light irradiation. Figure 8 reveals that no obvious decrease of the photocatalytic activity was observed after five cycles, suggesting the excellent photocatalytic stability of the S-M Ag_2_CrO_4_ sample in the reactions.

**Figure 9:**
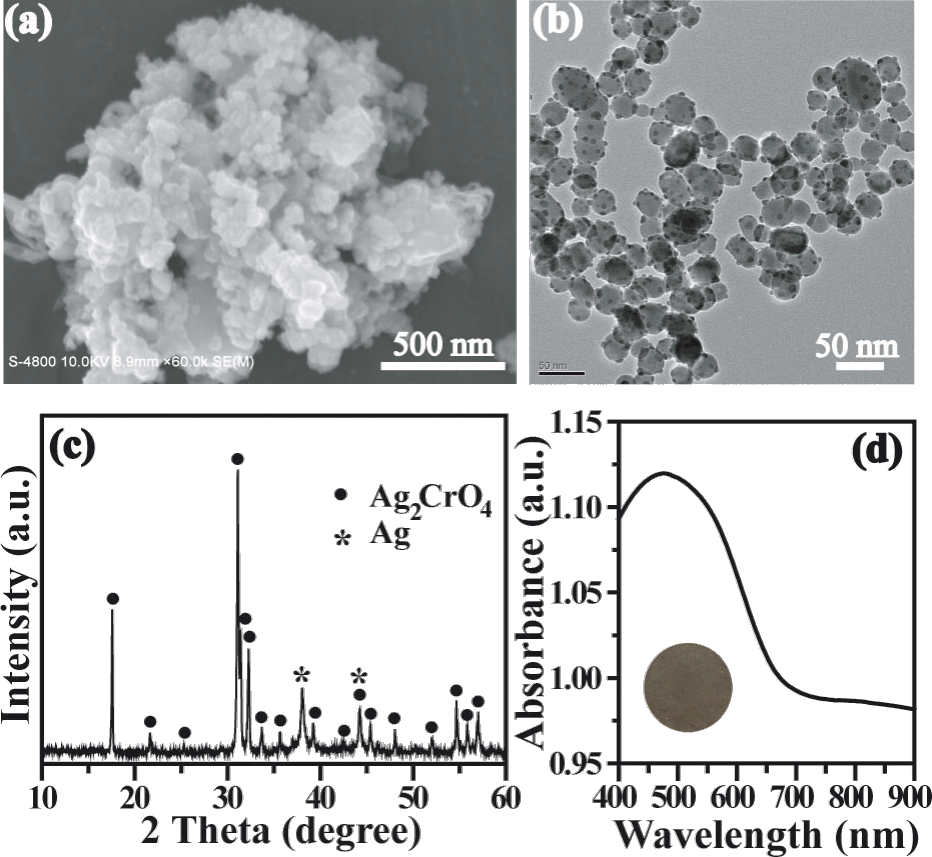
(a) SEM image, (b) TEM image, (c) XRD pattern, and (d) UV–visible spectrum of Ag_2_CrO_4_ after five circles of photocatalytic reaction.

To investigate the structure of Ag_2_CrO_4_ after five circles of the photocatalytic reaction, the corresponding SEM and TEM images, XRD pattern, and UV–vis diffuse reflectance spectrum were collected. Figure 9a and Figure 9b show that the overall morphology and average particle size of Ag_2_CrO_4_ were not changed significantly. However, some homogenously distributed Ag nanoparticles could be observed on the surface of Ag_2_CrO_4_ (Figure 9b). The existence of metallic Ag could be further demonstrated by the XRD pattern (Figure 9c), which displayed a new peak located at 2θ = 38.1° corresponding to the (111) plane of silver (JCPDS No. 65-2871). The UV–visible spectrum with the corresponding colour of Ag_2_CrO_4_ after five circles of photocatalytic reaction is displayed in Figure 9d. It was found that the absorbance intensity in the visible-light region largely increased, which could be ascribed to the darkened colour of Ag_2_CrO_4_ after photocatalysis, resulting from the silver nanoparticles [32]. These results indicate that Ag_2_CrO_4_ was partially reduced to metallic Ag and formed an Ag–Ag_2_CrO_4_ composite. However, the majority of Ag_2_CrO_4_ was still preserved, and the formed Ag particles may further promote the photocatalytic activity in terms of surface plasmon resonance [75–76] and electron-sink effect [30]. Therefore, the photocatalytic activity of Ag_2_CrO_4_ did not show obvious decrease after 5-circle reaction.

## Conclusion

In summary, a range of Ag_2_CrO_4_ photocatalysts are prepared by microemulsion, precipitation, and hydrothermal methods. The preparation methods exhibit a great influence on the structure, optical properties and photocatalytic activity of the Ag_2_CrO_4_ crystals. The sample prepared by microemulsion method has the smallest particle size, highest surface area, most efficient light absorption, and a blue-shifted absorption edge. Consequently, the microemulsion prepared Ag_2_CrO_4_photocatalyst shows the best activity in the photodegradation of a MB aqueous solution, because of the higher adsorption of MB molecules, shorter diffusion process of more photogenerated excitons, and stronger oxidation ability of the photogenerated holes. The current investigation may provide new insight into the effect of preparation methods on the structure and photocatalytic activity of photocatalysts. Future work may focus on the study of the visible-light photocatalytic mechanism and the stability promoting methods of Ag_2_CrO_4_. Moreover, it is also possible to investigate the photocatalytic activity of Ag_2_CrO_4_ toward the degradation of other organic pollutants under visible-light irradiation.

## Experimental

### Preparation of Ag_2_CrO_4_ photocatalysts

All chemicals were analytical grade and used without further purification. Deionized (DI) water was used in all experiments. The Ag_2_CrO_4_ photocatalysts were prepared by the microemulsion, precipitation, and hydrothermal methods. The corresponding samples were labeled as S-M, S-P and S-H, respectively, as listed in Table 1. The detailed experimental procedures for the preparation of the samples are described as follows:

**Sample S-M:** Under stirring, cyclohexane (16 mL), Triton X-100 (5.2 mL) and *n*-hexanol (3 mL) were mixed at room temperature. Then K_2_CrO_4_ aqueous solution (0.5 mL, 0.5 M) was dripped into the mixture under continuous stirring to form a clarified and transparent W/O reversed-micellar solution, followed by the dropwise addition of AgNO_3_ aqueous solution (0.5 mL, 0.25 M) and kept for 1 h under stirring. The resultant suspension was aged for 24 h.

**Sample S-P:** AgNO_3_ (1000 mL, 0.5 M) and K_2_CrO_4_ (1000 mL, 0.25 M) aqueous solutions were mixed together under vigorous stirring. The resultant suspension was then aged for 24 h.

**Sample S-H:** The sample prepared by precipitation method was loaded into a Teflon-lined stainless steel autoclave with a capacity of 100 mL, sealed, heated to and maintained at 160 °C for 16 h, and subsequently cooled to room temperature naturally.

All the samples were collected and washed by centrifugation–redispersion cycles with ethanol and water, and then dried at 70 °C for 4 h.

### Characterization

The XRD were recorded on an X-ray diffractometer (type HZG41BPC) with Cu Kα irradiation source at a scan rate (2θ) of 0.05°·s^−1^. The accelerating voltage and applied current were 40 kV and 80 mA, respectively. The morphology observation was carried out by SEM (S4800, Hitachi, Japan) at an accelerating voltage of 5 kV. TEM and HRTEM analysis were conducted by the transmission electron microscopy (JEM-2100F, JEOL, Japan) at an accelerating voltage of 200 kV. The DRS were taken with a UV–vis spectrophotometer (UV2550, Shimadzu, Japan). BaSO_4_ was used as a reflectance standard. The nitrogen adsorption and desorption isotherms were measured by using an ASAP 2020 system (Micromertitics instruments, USA) after the samples were degassed at 180 °C. The *S*_BET_ was determined by a multipoint BET method using the adsorption data in the relative pressure (*P*/*P*_0_) range of 0.05–0.3. The desorption data was used to determine the pore size distribution through the Barret–Joyner–Halenda (BJH) method. The nitrogen adsorption volume at *P*/*P*_0_ of 0.994 was used to determine the average pore size. Zeta potential was measured by electrophoretic light scattering with a zetasizer (Nano ZS90, Malvern, UK).

### Computational details

The DFT calculations were carried out to investigate the band structure and density of states (DOS) of Ag_2_CrO_4_ model by using the CASTEP Packages on the basis of the plane-wave-pseudo-potential approach [77–78]. Combined with ultrasoft pseudo-potentials, the Perdew–Burke–Ernzerhof (PBE) of generalized gradient approximation (GGA) was applied as the exchange–correlation function [79–80]. The plane-wave cut-off energy was set to be 500 eV, the Monkhorst–Pack *k*-point in the Brilliouin Zone to be 3 × 5 × 6, and the self-consistent field (SCF) convergence accuracy to be 1 × 10^−6^ eV/atom. For the geometric optimization, the convergence criteria were set as follows: 1 × 10^−5^ eV/atom for total energy, 0.03 eV/Å for maximum force, 0.05 GPa for maximum stress, and 1 × 10^−3^ Å for maximum displacement. The energy and geometry structure showed no obvious change when higher cut-off energy and more *k*-points were adopted. The electronic structure calculation was carried out by using the optimized geometric structure.

### Measurements of photocatalytic activity

The photocatalytic activity of the as-prepared samples was evaluated by the photocatalytic degradation of MB under visible-light irradiation in water at ambient temperature. The prepared photocatalysts (50 mg) were firstly dispersed into water in a reactor with a diameter of 7.0 cm and then dried at 80 °C for 4 h, giving rise to the formation of Ag_2_CrO_4_ films at the bottom of the reactor. MB aqueous solution (50 mL, 2.5 × 10^−5^ M) was added into the reactor and kept in the dark for 30 min to ensure an adsorption–desorption equilibrium prior to irradiation. A 300 W xenon arc lamp coupled with a UV cut-off filter (λ ≥ 400 nm), which was positioned 20 cm away from the reactor, was used as a visible-light source to drive the photocatalytic reaction. The concentration of MB was determined by a UV–vis spectrophotometer (UV2550, Shimadzu, Japan). After irradiation for every 15 min, the reaction solution was taken out to measure the concentration change of MB. As for the MB aqueous solution with low concentration, its photocatalytic degradation was a pseudo-fist-order reaction and its kinetics was expressed as [81–83]:

[3]
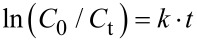


Where *k* is the apparent rate constant, *C*_0_ and *C*_t_ are the initial and reaction concentrations of MB, respectively.
